# Serum cobalamin and methylmalonic acid concentrations in juvenile dogs with parvoviral enteritis or other acute enteropathies

**DOI:** 10.1111/jvim.16736

**Published:** 2023-05-16

**Authors:** Michael Hung, Justin Heinz, Jӧrg M. Steiner, Jan Suchodolski, Jonathan Lidbury

**Affiliations:** ^1^ Department of Small Animal Clinical Sciences, School of Veterinary Medicine & Biomedical Sciences Texas A&M University College Station Texas USA

**Keywords:** acute enteropathy, canine parvovirus, cobalamin deficiency, diarrhea, juvenile

## Abstract

**Background:**

Low serum cobalamin concentrations have been associated with ileal malabsorption in dogs with chronic enteropathy. Increased serum methylmalonic acid (MMA) concentrations indicate cobalamin deficiency on a cellular level. Few studies have evaluated serum cobalamin concentrations or methylmalonic acid concentrations in juvenile dogs with parvoviral enteritis or nonparvoviral acute enteropathies.

**Objectives:**

Evaluate serum cobalamin and methylmalonic acid concentrations in juvenile dogs (6 weeks to 10 months old) with parvoviral enteritis or nonparvoviral acute enteropathy.

**Animals:**

Thirty‐one juvenile dogs with parvoviral enteritis, 29 dogs with nonparvoviral acute diarrhea (NPVAD), and 40 healthy juvenile control dogs.

**Methods:**

Single‐center, prospective, observational, cross‐sectional study. Serum cobalamin and, when sufficient serum was available, MMA concentrations were measured.

**Results:**

Most serum cobalamin concentrations were within the adult reference interval. Serum cobalamin concentrations in healthy dogs (median, 848 ng/L; range, 293‐1912 ng/L) were significantly higher than in dogs with parvoviral enteritis (*P* = .0002; median, 463 ng/L; range, <150‐10 000 ng/L) or dogs with NPVAD (*P* = .02; median, 528 ng/L; range, 160‐8998 ng/L). Serum MMA concentrations were not significantly different between groups (healthy dogs: median, 796 nmol/L; range, 427‐1933 nmol/L; parvoviral enteritis: median, 858 nmol/L; range, 554‐3424 nmol/L; NPVAD: median, 764 nmol/L; range, 392‐1222 nmol/L; *P* = .1).

**Conclusions and Clinical Importance:**

Juvenile dogs with parvoviral enteritis or NPVAD had lower serum cobalamin concentrations than healthy juvenile dogs. However, based on serum MMA concentrations cellular cobalamin deficiency was not apparent.

**Abbreviations**
CPV: canine parvovirus
MMA: methylmalonic acid
NPVAD: nonparvovirus acute diarrhea
RI: reference interval

## 1 INTRODUCTION

Cobalamin, also known as Vitamin B_12_, is a water‐soluble vitamin required for DNA synthesis and cellular energy production.^1^ Cobalamin deficiency is a well‐characterized phenomenon in dogs with chronic enteropathy.^2^, ^3^ Measurement of serum cobalamin concentration commonly is used as a diagnostic marker for ileal malabsorption in small animal medicine^4^ and generally is presumed to occur secondary to a chronic inflammatory enteropathy, but can also occur secondary to exocrine pancreatic insufficiency^5^ or possibly small intestinal dysbiosis.^6^ Oral or parenteral supplementation^7^, ^8^ often is recommended for patients with serum cobalamin concentrations <400 ng/L.^7^, ^9^ The reported prevalence of low serum cobalamin concentration in dogs with chronic enteropathies ranges from 19% to 38%.^2^, ^10^

Dogs affected by Imerslund‐Gräsbeck syndrome (a mutation resulting in selective cobalamin malabsorption in the ileum) can develop blood cell dyscrasias (e.g., neutropenia, nonregenerative anemia), central and peripheral neurologic signs, and gastrointestinal (GI) signs such as anorexia, vomiting, diarrhea, lethargy, and failure to thrive.^11^, ^12^ Although some of these signs exist in dogs with chronic enteropathies, improvement generally is attributed to resolving the underlying condition as opposed to supplementation alone. Weight gain and improvement in clinical outcome have been described in cats with parenteral administration of cobalamin in addition to other treatments.^13^

Cobalamin is an essential cofactor of the enzyme methylmalonyl‐CoA mutase that converts L‐methylmalonyl‐CoA to succinyl‐CoA.^2^ Therefore, free MMA accumulates in patients with cobalamin deficiency at the cellular level. Measurement of serum MMA concentration is thought to be a more accurate indicator of the body's cobalamin status because serum cobalamin concentrations may not reflect cellular concentrations.^2^, ^10^, ^14^ However, serum MMA concentration is not routinely determined because measurement of MMA in serum is very labor‐intensive and expensive.

Enteritis caused by canine parvovirus type 2 is the world's most common infectious disease of dogs.^15^ The virus typically is shed in young puppies from 6 weeks to 6 months of age, and the virus is difficult to eliminate from the environment.^15^, ^16^ The disease results in severe damage to the intestinal epithelial crypt cells and other rapidly dividing cells, such as hematopoietic progenitor cells.^17^ Clinical signs are typically gastrointestinal in origin, although septicemia from bacterial translocation^18^ and myocarditis^19^ also can be seen.

Given the destructive effects of parvovirus on the intestine, a deficiency in cobalamin because of ileal malabsorption is possible. If so, cobalamin supplementation theoretically could be beneficial in affected dogs. At the same time, other acute processes can cause intestinal disease in puppies, and may or may not have a similar effect on cobalamin status. Although a previous study identified decreased serum cobalamin concentrations in dogs with parvoviral enteritis compared to healthy dogs,^20^ serum MMA concentrations, and therefore cellular cobalamin status, were not investigated. Furthermore, the prevalence and severity of cobalamin deficiency in dogs of similar age with other acute enteropathies were not described.

Therefore, we designed a prospective study to investigate serum cobalamin and MMA concentrations in juvenile dogs with parvoviral enteritis, dogs with nonparvoviral acute diarrhea (NPVAD), and juvenile healthy control dogs. It was hypothesized that serum cobalamin concentrations in dogs with any enteropathy would be lower than in healthy dogs and would be associated with an increase in serum MMA concentrations, indicating cellular cobalamin deficiency.

## 2 MATERIALS AND METHODS

Ours was a single‐center, prospective, observational cross‐sectional study. Dogs between 6 weeks and 10 months of age that presented to the Texas A&M Veterinary Medical Teaching Hospital Small Animal Emergency Service or Primary Care service between October 2019 through June 2022 were eligible for inclusion. The study was approved by the Institutional Animal Care and Use Committee (2019‐0258). Written consent was provided by the owner of each dog before enrollment into the study.

Eligible dogs had to weigh at least 2 kg, have had at least 2 episodes of diarrhea with or without vomiting within the last 12 hours or diarrhea with or without vomiting or inappetence lasting over 24 hours, and a commercial, rapid, patient‐side canine parvovirus (CPV) SNAP ELISA (Idexx Laboratories, USA) performed at the facility or at a referring veterinarian's practice. These dogs then were divided into 2 groups. One group included dogs that tested positive on ELISA, called the CPV group. The other group consisted of dogs that tested negative on ELISA, called the nonparvovirus acute diarrhea (NPVAD) group. Fecal samples were required for inclusion into the latter group for fecal CPV PCR testing. Dogs that tested positive on PCR were considered part of the CPV group. Dogs that were diagnosed with a foreign body or intussusception after the study were excluded. Dogs with diarrhea lasting >3 weeks were excluded from the study. Age, breed, sex, reproductive status, vaccination status, and duration of reported clinical signs were recorded for each dog.

Clinically healthy dogs >2 kg between the ages of 6 weeks and 10 months that presented for routine vaccination or an elective surgical procedure were included as controls. Dogs in this group were considered healthy based on history and physical examination findings with no evidence of vomiting or diarrhea within the 72 hours before presentation. After sample collection, each dog was treated for its primary reason for presentation at the discretion of the attending clinician.

### 2.1 Sample processing

Whole blood was collected from each dog and stored in no‐additive collection tubes at 4°C within 24 hours of presentation. If not processed for cobalamin measurement within 8 hours, whole blood was centrifuged within 24 hours and serum stored at −20°C.

When required, fecal samples were collected using swab, digital rectal examination, or floor collection and stored in plastic containers at 4°C. Real‐time PCR was performed on the samples for CPV DNA as previously described.^21^

### 2.2 Assays

Serum cobalamin measurements were performed in all dogs within 48 hours of blood collection using a solid‐phase competitive chemiluminescent immunoassay (Vitamin B_12_, Immulite 2000; Siemens Healthcare Diagnostics). The working range was from 150 to 1000 ng/L, with a reference interval (RI) of 251 to 908 ng/L. When the cobalamin concentration exceeded the upper detection limit, the serum sample was diluted and rerun to obtain a numerical value. Results >10 000 ng/L were entered as 10 000 ng/L for statistical analysis. The surplus serum for MMA measurement was stored at −80°C. Serum MMA concentration was measured using the stable isotope dilution gas chromatography‐mass spectrometry method in batches as described previously.^2^ The RI for serum MMA in dogs previously was determined to be 415 to 1193 nmol/L.^2^ The lower and upper detection limits for the assay were 63 and 16 000 nmol/L, respectively.

### 2.3 Statistical methods

A power calculation showed that at least 22 dogs per group were needed to detect a difference of 200 ng/L between groups. This determination was made using a SD of 236 ng/L for serum cobalamin concentration,^22^ power of 0.8, and an alpha of 0.05.

Continuous data were assessed for normality using D'Agostino‐Pearson tests and visual inspection of q‐q plots. The data was nonparametric and is expressed as median (range). Differences in serum cobalamin and MMA concentrations between groups were assessed using Kruskal‐Wallis tests followed by Dunn's post‐test as appropriate. The correlation between serum cobalamin and MMA concentrations was assessed using Spearman's rank correlation coefficient. Statistical analysis was performed using a commercial software package (Prism v8, GraphPad, San Diego, California). Values of *P* < .05 were considered significant.

## 3 RESULTS

During this period, 102 dogs with acute GI signs were screened. One dog was excluded because it tested positive on SNAP CPV, but was negative on concurrent fecal PCR. This patient was treated for CPV and recovered but was excluded from the analysis because of the discordant test results. Another dog was excluded for diarrhea lasting >3 weeks. One hundred dogs were included in the analysis. No dogs were diagnosed with foreign bodies or intussusception. Forty healthy dogs also were enrolled.

### 3.1 Demographics

#### 3.1.1 CPV group

Thirty‐one dogs were enrolled in the CPV group. Twenty‐four dogs tested positive on a SNAP ELISA. Seven dogs tested negative on SNAP testing but tested positive on fecal PCR. All 7 dogs were reported to have previous vaccinations, ranging from receiving 1 vaccine 1 day before presentation (2 dogs) to finishing 3 rounds of vaccinations 2 months before (1 dog). Four of these dogs had clinical signs consistent with canine parvovirus (e.g., obtunded, leukopenic, severe diarrhea) and were hospitalized. The remaining 3 dogs were treated on an outpatient basis and were all doing well at the time of the follow‐up call regarding the positive PCR result. All 3 dogs had a parvovirus vaccine administered at least 7 days before.

One dog tested negative for both SNAP CPV and fecal PCR but tested positive on PCR run on jejunal tissue taken on necropsy. This dog was included as part of the CPV group.

Thirteen dogs presented with signs of inappetence in addition to reported diarrhea. Thirteen dogs had signs of vomiting, diarrhea, or inappetence lasting for >1 day before presentation, with the longest duration of signs being 4 days.

American Pit Bull Terriers (5) and mixed breeds (4) were the most common breeds represented. The remaining breed distribution was as follows: German Shepherd (3), Golden Retriever (3), Australian Shepherd (2), Doberman Pinscher (2), Labrador Retriever (2), Black Mouth Cur, Blue Heeler, Border Collie, Boxer, Catahoula, Great Dane, Great Pyrenees, Heeler mix, Miniature Poodle, and Walker Hound (1 each). Twelve females (9 intact, 3 spayed) and 19 males (18 intact, 1 castrated) were included. The median age was 3.5 months (range, 1.5‐9.0 months).

#### 3.1.2 NPVAD group

Twenty‐nine dogs were enrolled in the NPVAD group. Three dogs presented with signs of inappetence in addition to reported diarrhea. Twelve dogs had signs of vomiting, diarrhea, or inappetence lasting for >1 day before presentation, with the longest duration of signs being 3 weeks. This latter dog was diagnosed with a whipworm infection.

Mixed breed dogs (6) and Australian Shepherds (5) were the most common breeds represented. The remaining breed distribution was as follows: Blue Heeler (3), American Pit Bull Terrier (2), German Shepherd (2), Labrador Retriever (2), Shih Tzu (2), Anatolian Shepherd, Basset Hound, Border Collie, Maltese, Pembroke Welsh Corgi, Rottweiler, and Samoyed (1 each). Twelve females (10 intact, 2 spayed) and 17 males (15 intact, 2 castrated) were included. The median age was 4.8 months (range, 1.3‐10.0 months).

#### 3.1.3 Healthy group

Forty dogs were enrolled in the healthy group. Mixed breed dogs (7) and Golden Retrievers (6) were the most common breeds represented. The remaining breed distribution was as follows: French Bulldog (4), Labrador Retriever (3), Pug (2), Border Collie (2), English Bulldog (2), American Pit Bull Terrier, Australian Shepherd, Basset Hound, Bernese Mountain Dog, Bichon Frise, Catahoula, Chihuahua, Cocker Spaniel, German Short‐haired Pointer, Great Dane, Maltese, Pembroke Welsh Corgi, Pomeranian, and Standard Poodle (1 each). Nineteen females (18 intact, 1 spayed) and 21 males (20 intact, 1 castrated) were included. The median age was 5 months (range, 1.75‐10.25 months).

#### 3.1.4 Comparison among groups

**FIGURE 1:**
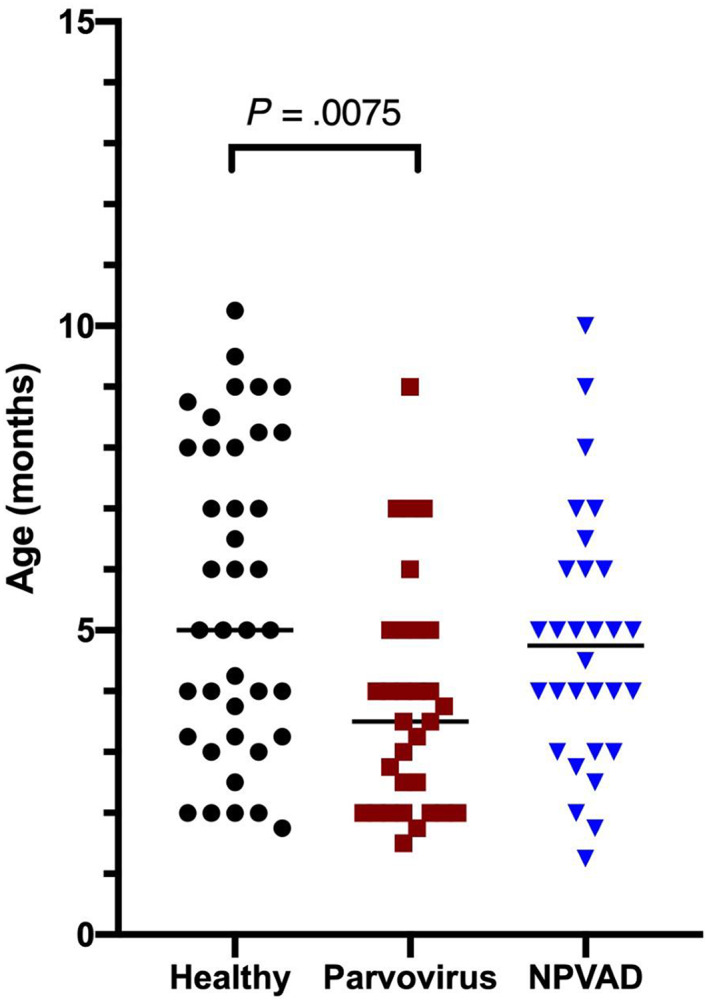
Age (months) distribution among the groups. The bar shows the median age for each group. Healthy control dogs were significantly older than dogs in the Parvovirus group.

No significant association between sex and groups of dogs was observed (*P* = .7). A significant difference in age among groups was found; healthy dogs were older than the CPV group (*P* = .008; Figure 1).

### 3.2 Serum cobalamin concentrations

**FIGURE 2:**
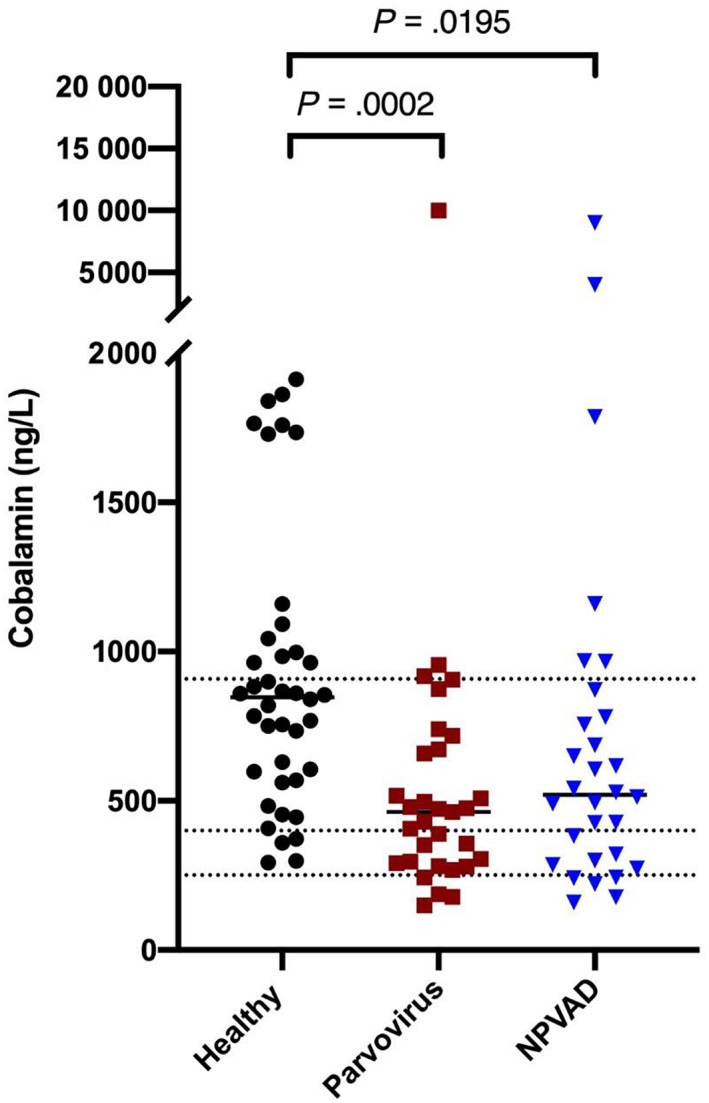
Serum cobalamin concentrations among the 3 groups. Note that the y‐axis is broken (0‐2000 ng/L and 5000‐20 000 ng/L). The top and bottom dashed lines represent the RI (251‐908 ng/L), with the center dashed line representing 400 ng/L. The black bars show the median serum cobalamin concentration for each group.

Serum cobalamin concentrations were compared between groups (Figure 2). The median serum concentration of cobalamin in the CPV group was 463 ng/L (range, <150‐10 000 ng/L). One dog in the CPV group had a serum cobalamin concentration > 10 000 ng/L. Thirteen dogs had serum cobalamin concentrations <400 ng/L. Of these, 5 dogs had cobalamin concentrations below the lower limit of the reference interval.

The median serum cobalamin concentration in the NPVAD group was 528 ng/L (range, 160‐8998 ng/L). One dog had a serum cobalamin concentration >4000 ng/L, but an exact value could not be determined because of insufficient sample volume for further dilution. Ten dogs had serum cobalamin concentrations <400 ng/L. Of these, 3 dogs had serum cobalamin concentrations below the lower limit of the reference interval.

The median concentration in the healthy group was 848 ng/L (range, 293‐1912 ng/L). Four dogs had serum cobalamin concentrations <400 ng/L. No dog had a serum cobalamin concentration below the lower limit of the reference interval.

Serum cobalamin concentrations were significantly different among all 3 groups (*P* = .0002). Most results were within the adult reference interval across all 3 groups. Serum cobalamin concentrations in the healthy dogs were significantly higher than in both the CPV group (*P* = .0002) and the NPVAD group (*P* = .02). However, no significant difference was found between the CPV and NPVAD groups (*P* = .8).

### 3.3 Serum MMA concentrations

**FIGURE 3:**
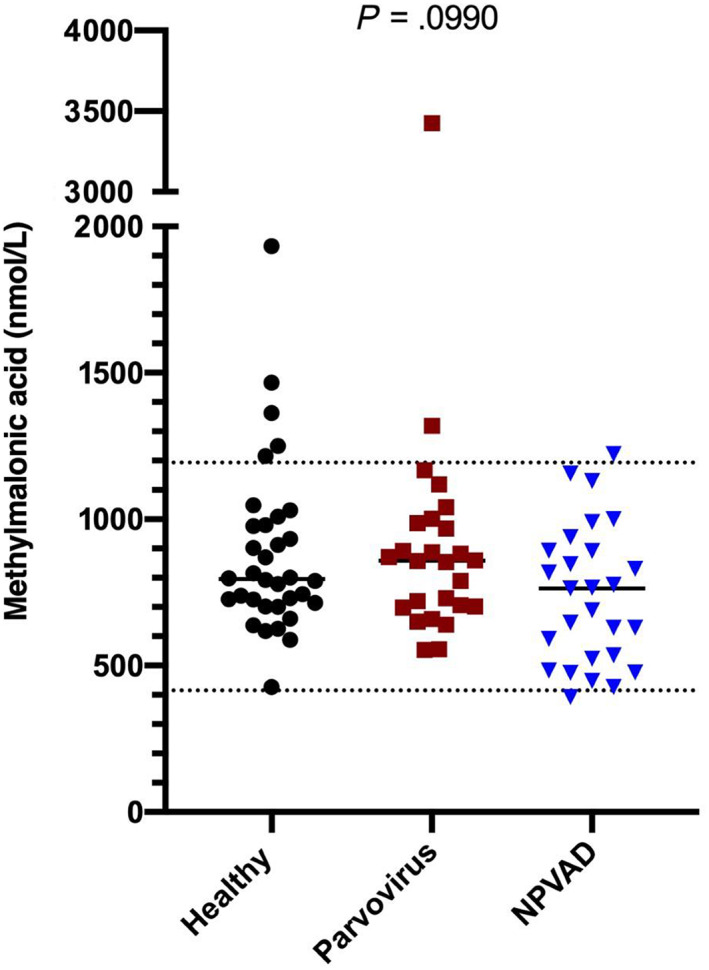
Serum MMA concentrations among the 3 groups. Note that the y‐axis is broken (0‐2000 nmol/L and 3000‐4000 nmol/L). The top and bottom dashed lines represent the limits of the RI (415‐1193 nmol/L). The black bars show the median MMA concentrations for each group.

Available serum MMA concentrations were compared among all groups (Figure 3). Eighty‐seven dogs had sufficient serum available for analysis. Fourteen dogs (5 parvoviral enteritis, 3 NPVAD, and 6 healthy dogs) did not have sufficient serum for MMA measurement. None of these dogs had a serum cobalamin concentration below the lower limit of the reference interval. Six of these dogs had serum cobalamin concentrations <400 ng/L (3 from the CPV group, 1 from the NPVAD group, and 2 from the healthy group).

Serum MMA concentrations were not significantly different among the groups (*P* = .1). The median serum MMA concentration in the CPV group was 858 nmol/L (range, 554‐3424 nmol/L). The median serum MMA concentration in the NPVAD group was 764 nmol/L (range, 392‐1222 nmol/L). The median concentration in the healthy group was 796 nmol/L (range, 427‐1933 nmol/L). The single dog with a serum MMA concentration of 3424 nmol/L was a Labrador Retriever.

Eight dogs had serum MMA concentrations above the upper limit of the reference interval (1193 nmol/L): 2 from the CPV group, 1 from the NPVAD group, and 5 from the healthy group.

### 3.4 Correlation between serum cobalamin and MMA concentrations

**FIGURE 4:**
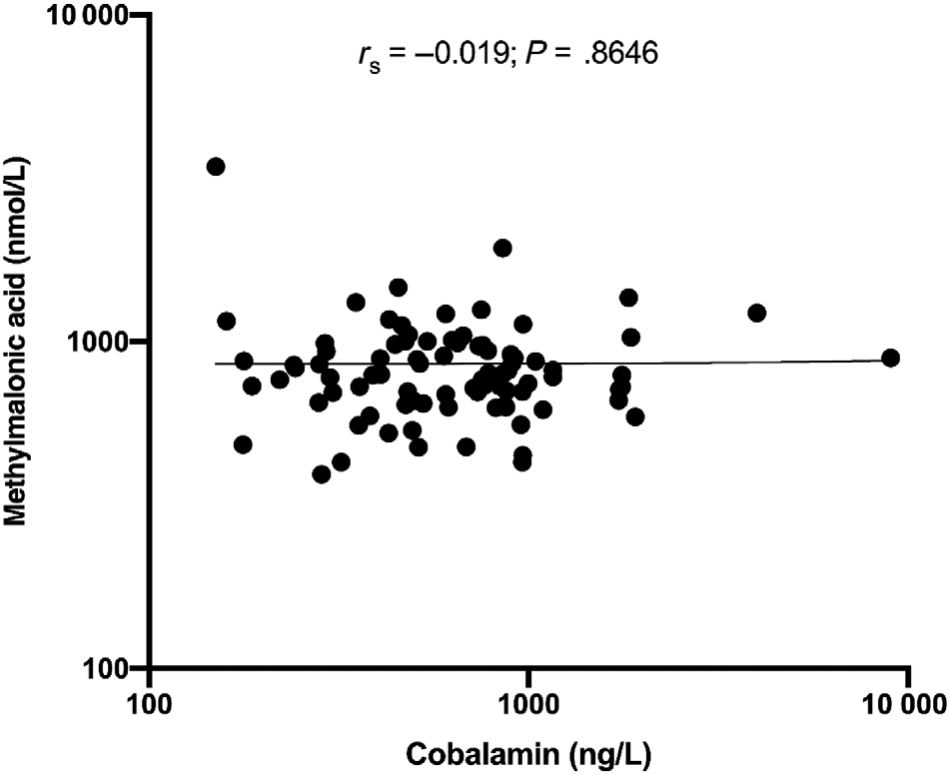
Methylmalonic acid concentrations plotted (y axis) plotted against cobalamin concentrations (x axis). Note that both axes are plotted on a logarithmic scale. The solid line indicates the best fit. No correlation between methylmalonic acid concentrations and cobalamin concentrations was found (Spearman's coefficient: −0.019, 95% confidence interval: −0.236 to 0.194, *P* = .8646).

No significant correlation was found between serum cobalamin and MMA concentrations (Spearman's rho: −0.2; 95% confidence interval [CI] −0.2356 to 0.1938; Figure 4).

## 4 DISCUSSION

We showed that healthy juvenile dogs had higher serum cobalamin concentrations than dogs with CPV enteritis or NPVAD. However, serum MMA concentrations did not show any significant differences among groups, suggesting no alteration in cellular cobalamin status in dogs with CPV or NPVAD.

Cobalamin is absorbed primarily by the ileal enterocytes via the cobalamin‐intrinsic factor complex.^23^, ^24^ As such, mucosal disease,^25^ lack of intrinsic factor,^5^ and small intestinal dysbiosis^6^ can be responsible for cobalamin malabsorption and resultant hypocobalaminemia. Canine parvoviral enteritis is known to cause malabsorption, increased intestinal permeability,^26^ and intestinal dysbiosis.^27^, ^28^ An acute enteropathy could cause similar changes, as observed in acute hemorrhagic diarrhea syndrome.^29^ Any of these aforementioned causes, on their own or together, could result in decreased serum cobalamin concentrations. However, substantial body stores of cobalamin are found in the liver, and thus serum concentrations of cobalamin may not reflect acute cobalamin malabsorption. Therefore, whether acute gastrointestinal disease could cause cobalamin deficiency is uncertain.

Our results mirror a previous study in which dogs with CPV had lower serum cobalamin concentrations than healthy dogs.^20^ The previous study did not measure MMA or any other markers for cellular cobalamin deficiency, such as homocysteine. In our study, no significant difference in serum MMA concentrations was found among groups, and no correlation between serum concentrations of cobalamin and MMA was found. When present, increases in serum MMA concentrations generally were mild in comparison to the reference interval for adult dogs, with the exception of 1 dog from the CPV group. Six dogs with serum MMA concentrations above the upper limit of the reference interval did not have a serum cobalamin concentration <400 ng/L. In studies of dogs with chronic enteropathies, a negative correlation between serum cobalamin and MMA concentrations has been described, suggesting that cellular cobalamin deficiency is related to serum concentrations.^2^ The lack of correlation between serum cobalamin and MMA concentrations in these juvenile dogs with acute enteropathies suggests that decreased serum cobalamin concentrations do not reflect cellular cobalamin deficiency. One potential explanation is that in dogs with severe acute enteropathies, hypocobalaminemia may occur secondary to the loss of transcobalamin and other cobalamin‐binding proteins from the blood through a “leaky” gut wall, causing rapid depletion of cobalamin from the serum. This situation could lead to an acute decrease in serum cobalamin concentration in the absence of the depletion of hepatic stores. It is also possible that as the acute enteropathy resolves, serum cobalamin concentrations will naturally return to normal without supplementation or a cellular deficiency ever developing. Therefore, we do not recommend the routine supplementation of cobalamin in juvenile dogs with acute enteropathies, even in those with hypocobalaminemia. However, because 1 dog from the CPV group with undetectable cobalamin had a marked increase in serum MMA concentration (3424 nmol/L), cellular cobalamin deficiency may occur in some juvenile dogs with acute enteropathies. This dog was not a reported breed affected by Imerslund‐Gräsbeck syndrome, had clinical signs (e.g., diarrhea, vomiting, inappetence, lethargy) lasting 24 hours at the time of presentation, and was treated on an outpatient basis because of owner financial limitations. Future longitudinal studies assessing serum cobalamin and MMA concentrations as well as outcome measures in dogs with acute enteropathies are needed. Because dogs with CPV are more likely to develop chronic enteropathies later in life,^30^ the association between hypocobalaminemia in CPV enteritis and subsequent development of chronic enteropathy also should be investigated.

Our study had some limitations. First, although we recruited healthy dogs from the same general age group (6 weeks to 10 months) as the diseased dogs, a small but significant difference in age was found between normal dogs and dogs with CPV. Serum cobalamin concentrations in human neonates are different than those of adults. This observation could explain the difference in serum cobalamin concentrations between the healthy dogs and dogs with CPV. A significant difference however was not found between NPVAD dogs and other groups, nor was an overall difference noted between serum cobalamin concentrations in juvenile dogs and adult reference ranges noted in a previous study.^20^ Also, we did not distinguish among types of diarrhea. Dogs with colitis would be assumed not to have a problem with cobalamin absorption and this difference could have affected the serum cobalamin concentrations seen in the NPVAD group. Dogs with colitis were not excluded from the study, because dogs had a limited evaluation and the diagnosis for most of these dogs was nonspecific. A detailed history of diarrhea was not required for enrollment and thus localizing clinical signs reported by the client were not always recorded. Other contributing causes for hypocobalaminemia were not excluded. Additional theoretical causes for hypocobalaminemia could involve exocrine pancreatic insufficiency,^9^ small intestinal bacterial overgrowth,^6^ or a vegetarian diet.^31^ Characterization of the fecal microbiome was not performed in our study. A thorough dietary history was not a criterion for enrollment, and was not available for all dogs. However, no owners reported feeding a vegetarian diet when a dietary history was available (58/100 dogs). Exocrine pancreatic insufficiency also was not excluded because it would be unlikely given our patient demographic.

Also, we only evaluated serum MMA concentrations as a marker of cellular cobalamin deficiency. Methylmalonic acid is excreted by the kidneys, and serum MMA concentrations are thought to be more stable and have a 40‐fold higher concentration in urine.^22^ Urine sample acquisition would have been difficult given the inconsistency in dogs urinating in the hospital and because clients may be unwilling to have their healthy animal undergo cystocentesis without a clinical indication. Other studies also have evaluated serum MMA concentrations in dogs.^9^

Other causes for increased serum MMA concentrations include hypovolemia, renal insufficiency, and genetic disorders.^10^ It is possible that the serum MMA concentrations above the upper limit of the adult reference interval in the 8 affected (parvovirus or NPVAD) dogs may have been secondary to hypovolemia or renal insufficiency. Six dogs had normal renal function test results, whereas no blood test results were obtained in the remaining 2 dogs. Only 1 healthy dog with increased serum MMA concentration did not have concurrent renal function tests evaluated, but interestingly this dog did not have a low serum cobalamin concentration.

Dogs that tested positive on fecal PCR were placed in the CPV group. However, recent vaccination can cause false positives and dogs also can test positive on fecal PCR without clinical signs consistent with parvoviral infection.^32^, ^33^, ^34^ Seven dogs that were PCR positive were in this group, with 3 having a history of recent vaccination (within 1 month). None of these dogs had a low serum cobalamin concentration, and thus could not have led to type I statistical error by their inclusion.

In conclusion, at the time of presentation, juvenile dogs with parvoviral enteritis or NPVAD had lower serum cobalamin concentrations than healthy juvenile dogs. However, based on serum MMA concentrations, this finding was not associated with cellular cobalamin deficiency. Future studies longitudinally assessing serum cobalamin and MMA concentrations as well as clinical outcome in similar groups of dogs are needed.

## CONFLICT OF INTEREST DECLARATION

Drs Lidbury, Steiner, and Sucholdolski are employed by the Texas A&M Gastroenterology (GI) Laboratory, which offers measurement of serum cobalamin and methylmalonic acid concentrations and detection of parvovirus DNA in feces by PCR on a fee‐for‐service basis.

## OFF‐LABEL ANTIMICROBIAL DECLARATION

Authors declare no off‐label use of antimicrobials.

## INSTITUTIONAL ANIMAL CARE AND USE COMMITTEE (IACUC) OR OTHER APPROVAL DECLARATION

Approved by Texas A&M IACUC, number 2019‐0258.

## HUMAN ETHICS APPROVAL DECLARATION

Authors declare human ethics approval was not needed for this study.

## References

[1] Green R , Allen LH , Bjørke‐Monsen AL , et al. Vitamin B_12_ deficiency. Nat Rev Dis Primers. 2017;3(1):17040. 28660890 10.1038/nrdp.2017.40

[2] Berghoff N , Parnell NK , Hill SL , Suchodolski JS , Steiner JM . Serum cobalamin and methylmalonic acid concentrations in dogs with chronic gastrointestinal disease. Am J Vet Res. 2013;74(1):84‐89. 23270350 10.2460/ajvr.74.1.84

[3] Allenspach K , Wieland B , Gröne A , Gaschen F . Chronic enteropathies in dogs: evaluation of risk factors for negative outcome. J Vet Intern Med. 2007;21(4):700‐708. 17708389 10.1892/0891-6640(2007)21[700:ceideo]2.0.co;2

[4] Suchodolski JS , Steiner JM . Laboratory assessment of gastrointestinal function. Clin Tech Small Anim Pract. 2003;18(4):203‐210. 14738200 10.1016/S1096-2867(03)00075-6

[5] Batchelor DJ , Noble PJM , Taylor RH , Cripps PJ , German AJ . Prognostic factors in canine exocrine pancreatic insufficiency: prolonged survival is likely if clinical remission is achieved. J Vet Intern Med. 2007;21(1):54‐60. 17338150 10.1892/0891-6640(2007)21[54:pficep]2.0.co;2

[6] Rutgers HC , Batt RM , Elwood CM , Lamport A . Small intestinal bacterial overgrowth in dogs with chronic intestinal disease. J Am Vet Med Assoc. 1995;206(2):187‐193. 7751219

[7] Toresson L , Steiner JM , Razdan P , et al. Comparison of efficacy of oral and parenteral cobalamin supplementation in normalising low cobalamin concentrations in dogs: a randomised controlled study. Vet J. 2018;232:27‐32. 29428088 10.1016/j.tvjl.2017.12.010

[8] Toresson L , Steiner JM , Spodsberg E , et al. Effects of oral versus parenteral cobalamin supplementation on methylmalonic acid and homocysteine concentrations in dogs with chronic enteropathies and low cobalamin concentrations. Vet J. 2019;243:8‐14. 30606444 10.1016/j.tvjl.2018.11.004

[9] Chang CH , Lidbury JA , Suchodolski JS , Steiner JM . Effect of oral or injectable supplementation with cobalamin in dogs with hypocobalaminemia caused by chronic enteropathy or exocrine pancreatic insufficiency. J Vet Intern Med. 2022;36(5):1607‐1621. 36054643 10.1111/jvim.16528 PMC9511088

[10] Kather S , Grützner N , Kook PH , Dengler F , Heilmann RM . Review of cobalamin status and disorders of cobalamin metabolism in dogs. J Vet Intern Med. 2020;34(1):13‐28. 31758868 10.1111/jvim.15638 PMC6979111

[11] Fyfe JC , Hemker SL , Venta PJ , Stebbing B , Giger U . Selective intestinal cobalamin malabsorption with proteinuria (Imerslund‐Gräsbeck syndrome) in juvenile beagles. J Vet Intern Med. 2014;28(2):356‐362. 24433284 10.1111/jvim.12284 PMC3959579

[12] Sancho IM , Holmes A , Adamantos S . Imerslund‐Grasbeck syndrome in a cross‐breed dog. J Small Anim Pract. 2021;62(8):709‐711. 33022748 10.1111/jsap.13239

[13] Ruaux CG , Steiner JM , Williams DA . Early biochemical and clinical responses to cobalamin supplementation in cats with signs of gastrointestinal disease and severe hypocobalaminemia. J Vet Intern Med. 2005;19(2):155‐160. 15822558 10.1892/0891-6640(2005)19<155:ebacrt>2.0.co;2

[14] Langan RC , Goodbred AJ . Vitamin B_12_ deficiency: recognition and management. Am Fam Physician. 2017;96(6):384‐389. 28925645

[15] Mylonakis ME , Kalli I , Rallis TS . Canine parvoviral enteritis: an update on the clinical diagnosis, treatment, and prevention. Vet Med (Auckl). 2016;7:91‐100. 30050842 10.2147/VMRR.S80971 PMC6053044

[16] Mazzaferro EM . Update on canine parvoviral enteritis. Vet Clin North Am Small Anim Pract. 2020;50(6):1307‐1325. 32891439 10.1016/j.cvsm.2020.07.008 PMC7467068

[17] Pollock R . Experimental canine parvovirus infection in dogs. Cornell Vet. 1982;72(2):103‐119. 6211333

[18] Turk J , Miller M , Brown T . *Coliform septicemia* and pulmonary disease associated with canine parvoviral enteritis: 88 cases (1987‐1988). J Am Vet Med Assoc. 1990;196(5):771‐773. 2155191

[19] Robinson WF , Huxtable CRR , Pass DA , Howell JMC . Clinical and electrocardiographic findings in suspected viral myocarditis of pups. Aust Vet J. 1979;55(8):351‐355. 533485

[20] Engelbrecht M , Botha WJ , Pazzi P , McClure V , Hooijberg E . Serum cobalamin concentrations in dogs infected with canine parvoviral enteritis. J Am Vet Med Assoc. 2022;260(7):1‐8. 10.2460/javma.21.05.0240 35113794

[21] Decaro N , Elia G , Martella V , Desario C . A real‐time PCR assay for rapid detection and quantitation of canine parvovirus type 2 in the feces of dogs. Vet Microbiol. 2005;105(1):19‐28. 15607080 10.1016/j.vetmic.2004.09.018

[22] Lutz S , Sewell AC , Biggler B , et al. Serum cobalamin, urine methylmalonic acid, and plasma total homocysteine concentrations in Border Collies and dogs of other breeds. Am J Vet Res. 2012;73(8):1194‐1199. 22849680 10.2460/ajvr.73.8.1194

[23] Batt RM , Horadagoda NU . Gastric and pancreatic intrinsic factor‐mediated absorption of cobalamin in the dog. Am J Physiol. 1989;257(3 Pt 1):G344‐G349. 2782408 10.1152/ajpgi.1989.257.3.G344

[24] Marcoullis G , Rothenberg SP . Intrinsic factor‐mediated intestinal absorption of cobalamin in the dog. Am J Physiol. 1981;241(4):G294‐G299. 7315968 10.1152/ajpgi.1981.241.4.G294

[25] Procoli F , Mõtsküla PF , Keyte SV , Priestnall S , Allenspach K . Comparison of histopathologic findings in duodenal and ileal endoscopic biopsies in dogs with chronic small intestinal enteropathies. J Vet Intern Med. 2013;27(2):268‐274. 23398168 10.1111/jvim.12041

[26] Mohr AJ , Leisewitz AL , Jacobson LS , Steiner JM , Ruaux CG , Williams DA . Effect of early enteral nutrition on intestinal permeability, intestinal protein loss, and outcome in dogs with severe parvoviral enteritis. J Vet Intern Med. 2003;17(6):791‐798. 14658714 10.1111/j.1939-1676.2003.tb02516.x PMC7166426

[27] Park JS , Guevarra RB , Kim BR , et al. Intestinal microbial dysbiosis in beagles naturally infected with canine parvovirus. J Microbiol Biotechnol. 2019;29(9):1391‐1400. 31434168 10.4014/jmb.1901.01047

[28] Turk J , Fales W , Miller M , et al. Enteric *Clostridium perfringens* infection associated with parvoviral enteritis in dogs: 74 cases (1987‐1990). J Am Vet Med Assoc. 1992;200(7):991‐994. 1315727

[29] Heilmann RM , Guard MM , Steiner JM , Suchodolski JS , Unterer S . Fecal markers of inflammation, protein loss, and microbial changes in dogs with the acute hemorrhagic diarrhea syndrome (AHDS). J Vet Emerg Crit Care (San Antonio). 2017;27(5):586‐589. 28771910 10.1111/vec.12636

[30] Kilian E , Suchodolski JS , Hartmann K , Mueller RS , Wess G , Unterer S . Long‐term effects of canine parvovirus infection in dogs. PLoS One. 2018;13(3):e0192198. 29547647 10.1371/journal.pone.0192198 PMC5856261

[31] Rizzo G , Laganà AS , Rapisarda AM , et al. Vitamin B_12_ among vegetarians: status, assessment and supplementation. Nutrients. 2016;8(12):767. 27916823 10.3390/nu8120767 PMC5188422

[32] Decaro N , Crescenzo G , Desario C , et al. Long‐term viremia and fecal shedding in pups after modified‐live canine parvovirus vaccination. Vaccine. 2014;32(30):3850‐3853. 24793948 10.1016/j.vaccine.2014.04.050 PMC7115601

[33] Meggiolaro MN , Ly A , Rysnik‐Steck B , et al. MT‐PCR panel detection of canine parvovirus (CPV‐2): vaccine and wild‐type CPV‐2 can be difficult to differentiate in canine diagnostic fecal samples. Mol Cell Probes. 2017;33:20‐23. 28254505 10.1016/j.mcp.2017.02.007 PMC7125668

[34] Sykes JE . Chapter 14—Canine parvovirus infections and other viral enteritides. In: Sykes JE , ed. Canine and Feline Infectious Diseases. Saint Louis, MO: W.B. Saunders; 2014:141‐151.

